# Comparative diagnostic imaging in giant African land snails (*Achatinidae*)

**DOI:** 10.3389/fvets.2023.1223784

**Published:** 2023-10-03

**Authors:** Michaela Gumpenberger, Silvana Schmidt-Ukaj, Stephan Handschuh

**Affiliations:** ^1^Diagnostic Imaging, University of Veterinary Medicine Vienna, Vienna, Austria; ^2^Service for Birds and Reptiles, Small Animal Internal Medicine, Department for Companion Animals and Horses, University of Veterinary Medicine Vienna, Vienna, Austria; ^3^VetCore, University of Veterinary Medicine Vienna, Vienna, Austria

**Keywords:** snail, radiography, sonography, computed tomography, contrast medium

## Abstract

Giant African land snails (GALS) have become increasingly popular, for example, as pets or in kindergartens in Europe, but little is known about their clinically relevant anatomy, diseases, or further details in diagnostic imaging. The present study focuses on the techniques and image interpretation of radiography, computed tomography, and sonography in GALS. The aim of the study is to find the most appropriate imaging tool to visualize the various organs within the mantle cavity (also known as visceral mass) in GALS. The detailed anatomy of GALS is presented with numerous figures of the different imaging techniques. The sensory organs and nervous system will not be part of the present study.

## 1. Introduction

Giant African land snails (GALS) of the family *Achatinidae* are terrestrial snails, of which there are about 23,000 species worldwide (1). *Achatinidae* are the largest terrestrial snails in the world; they breathe with a lung, are mainly herbivores, and live for at least 10 years or more (2). These snails are considered crop pests in most countries, serve as a delicacy and protein source for humans in others, or are used as pet food, mainly for reptiles (2–5). However, they are quite popular pets in Austria, may accompany agitated children for reassurance, and can be used to motivate elderly people and those with disabilities (6, 7). They even support wound treatment and cosmetics due to their unique slime (8).

Dedicated owners are now seeking veterinarian support for their ill mollusks. There is little literature available about the anatomy and diagnostic imaging of snails and slugs, especially GALS, for veterinarians. A simple overview of the anatomy of *Lissachatina fulica* was provided by Benthem Jutting in 1951 (9). More recently, Cooper (1991) wrote a general overview of snails, including anatomy and physiology, for veterinarians (10). Ghose described in detail the alimentary and reproductive system of snails in 1963 (11, 12), while more recent studies focus only on the differences in the reproductive tract in single snail species (13–15).

The benefit of radiographs is mentioned in the literature for evaluation of cracks in the shell or even the use of contrast medium for visualization of the gastrointestinal tract in invertebrates (16). However, hardly any further or more detailed information about the procedure or radiographic anatomy is provided (17–19). The authors only mention that feeding a preferred dietary item, such as fruit, containing barium is used (17, 20).

Ultrasonography is described as a useful tool in the examination of GALS (19). Water is preferred as a coupling medium instead of ultrasound gel which seems to irritate GALS. It is stated that a small amount of water is sufficient because the copious mucus secreted by the gastropod foot serves as a natural coupling gel. Small 7.5–10 MHz curvilinear transducers have been used to visualize the oral radula, pharynx, and cranial digestive tract as well as developed eggs and the origin of prolapsed organs (19). Interestingly, it is recommended to sedate the snails to reduce movement artifacts. Nollens described the successful detection of shell lesions in live abalone with radiography and sonography (21).

The use of micro CT for illustration of the anatomy (especially the shell, though rarely the inner organs) of dead specimens has been demonstrated already (22–28). The animals were mostly stained with iodine to guarantee sufficient soft tissue differentiation. Rare studies describe micro CT examinations of tiny living snails for biological studies; these hardly serve the needs of larger patients (29). A single striking MRT image was used to point out the need for anesthesia in some imaging techniques in one paper, but no information about the imaging procedure or anatomic details was given (30). A more recent study for malacological research provides a very detailed insight into the technique and resulting images in CT and MRT in various aquatic mollusks, *in vivo* as well as *ex vivo* (31). These studies were mainly intended to gather structural information for zoological research.

The authors could not find more accurate studies about any diagnostic imaging procedures in living terrestrial snails that would provide appropriate information about pet snails for veterinarians. Therefore, the aim of the present prospective, descriptive, anatomic, and comparative imaging study was (1) to adapt and evaluate radiographic, computed tomographic (CT), and sonographic procedures for and in living land snails, (2) to describe the radiographic, sonographic and CT anatomy and appearance of the inner organs in GALS, and finally (3) to find the most appropriate imaging tool to visualize the various organs within the mantle cavity (also known as visceral mass) in GALS. The sensory organs and nervous system will not be part of the present study.

## 2. Materials and methods

### 2.1. Imaging procedures

**Figure 1:**
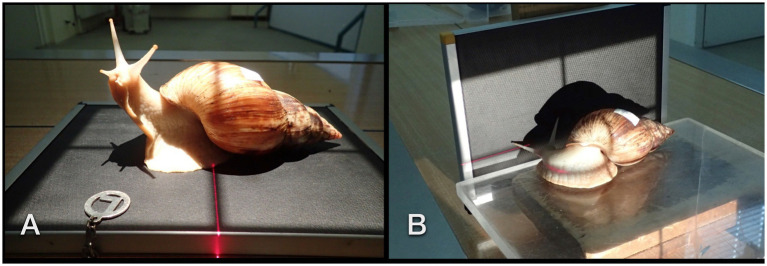
Dorsoventral **(A)** and lateral **(B)** radiographic examination in a GALS. The animal was curiously exploring its shadow and the light source.

In total, six individuals (two *L. fulicula* and four *L. albopicta albicans*), aged an estimated 3 to 9 years (according to the owners, depending on the time of obtainment and size of the animals) and weighing 85 to 223 g, received plain radiographs from the dorsoventral (DV, vertical beam, Figure 1A), laterolateral (LL, Figure 1B), and craniocaudal (CC, both horizontal beam technique) views using 60 kV and 2.5 mAs, a film focus distance of 50 cm, and a digital mammography cassette system (Kodak DirectView PQ storage phosphor screen/cassette, Carestream, Health Inc., Rochester, NY, USA).

**Figure 2:**
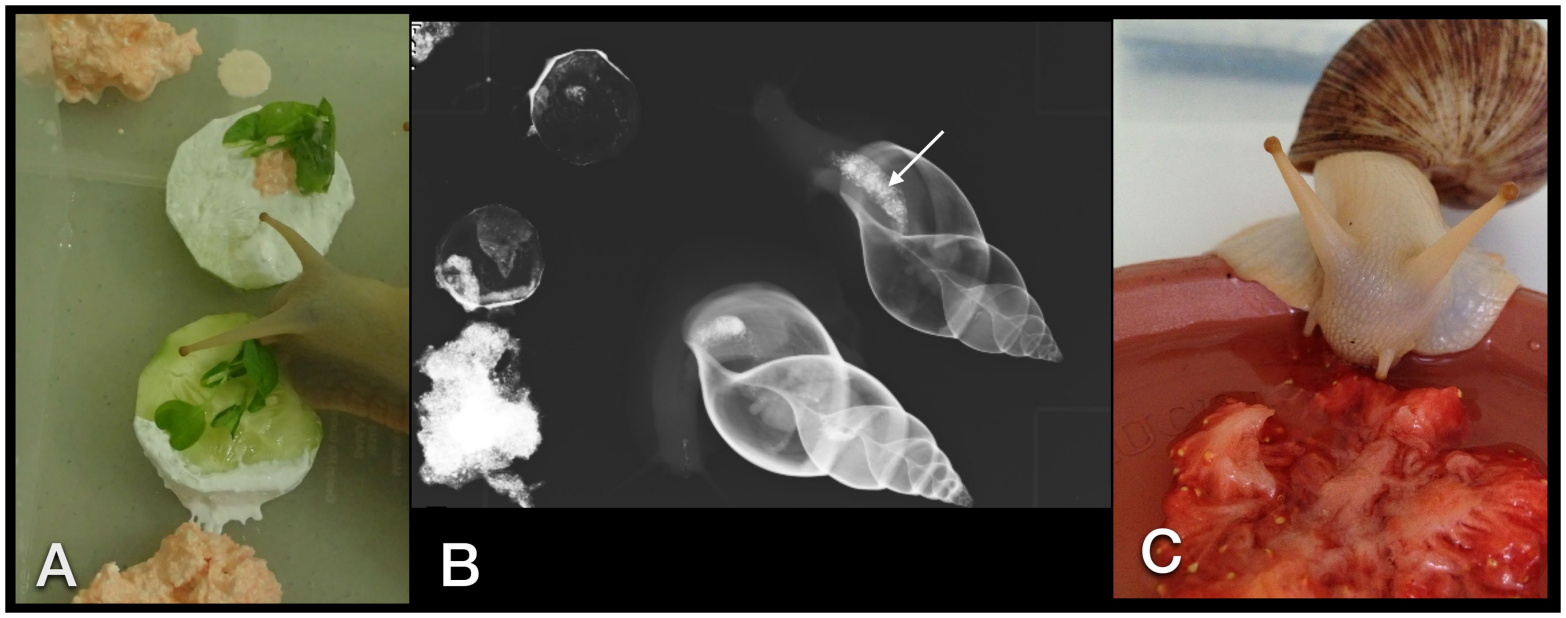
GALS feeding enthusiastically on a cucumber dipped in barium sulfate paste and herbs **(A)**. Rasped carrots mixed with contrast medium were placed alongside the cucumber. Overview dorsoventral radiograph of the snails that have ingested some bites of contrast medium and food **(B)**. Note the already enhanced crop (arrow). **(C)** A strawberry crushed with an iodine contrast medium was readily accepted as well.

Four snails were offered rasped cucumbers and carrots mixed with barium sulfate paste (Figures 2A,B) in the morning. The snails could feed on this meal for 10 h but did so only for up to 2 h. One other snail was offered a squashed strawberry floating in a lukewarm iodinated contrast medium (Unilux®, 370 mg iodine/ml; Figure 2C). Dorsoventral and LL radiographs were then repeated 15 min, 25 min, 1 h, 2 h, 3 h, 3,5 h, 4 h, 5 h, 6 h, 10 h, 11.5 h, 13 h, 14.5 h, 16 h, 21 h, 22 h, 24 h, 25 h, 33 h, 36 h, and 48 h after the animals started to feed. As soon as the contrast medium highlighted the gastrointestinal tract, one snail was selected for fluoroscopy. Fluoroscopy took place in ventral recumbency in a perpendicular beam at first. Then the animal was attached to the side of a plastic box for a CC and LL view (in a perpendicular beam) and, therefore, different visualizations of cardiac action. No restraint was necessary.

**Figure 3:**
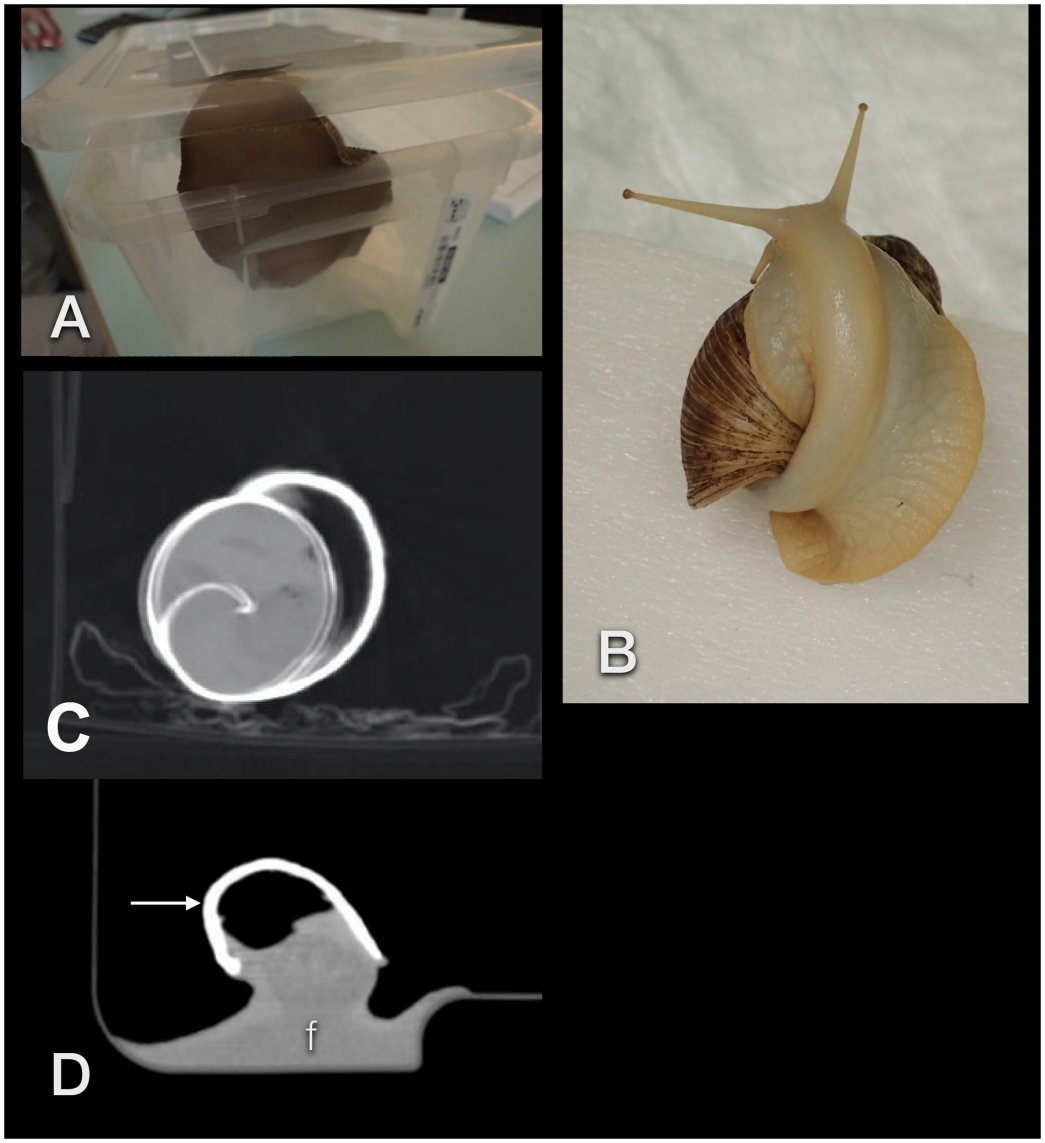
Unexpected events when performing CTs in GALS. The animals may easily lift the lid of a box **(A)** or climb onto their own shell **(B)**, thereby positioning themselves improperly. Movement artifact in an accelerated snail **(C)**. These patients may squeeze their feet (f) smoothly into any corner, resulting in an extraordinary, often awkward body shape **(D)**. The arrow points to the shell.

For CT examinations, all six individuals were placed, conscious, either on a Styrofoam block or in a plastic box (depending on the “temperament” and curiosity of the individual, Figures 3A–D) and positioned perpendicular to the gantry. Sagittal scans with a 16-slice helical CT (Siemens Somatom Emotion, Vienna, Austria) were performed using 80 m As, 130 kV, rotation time 1.5 s, pitch 0.8, slice thickness 0.6 mm, and a matrix of 512×512. Images were reconstructed with soft tissue (“mediastinum”) and a sharp bony kernel. The evaluation was done for modified soft tissue and bony window in a way that the examiner felt comfortable with differentiating the various organs. Four animals that were fed contrast medium for radiographs underwent multiple CT examinations after 1 h, 2.5 h, 5.5 h, 11.5 h, 24.5 h, 48 h, 52 h, 76 h, and, in one animal that drank iodine, additionally after 13 days. In total, 33 CT studies took place.

**Figure 4:**
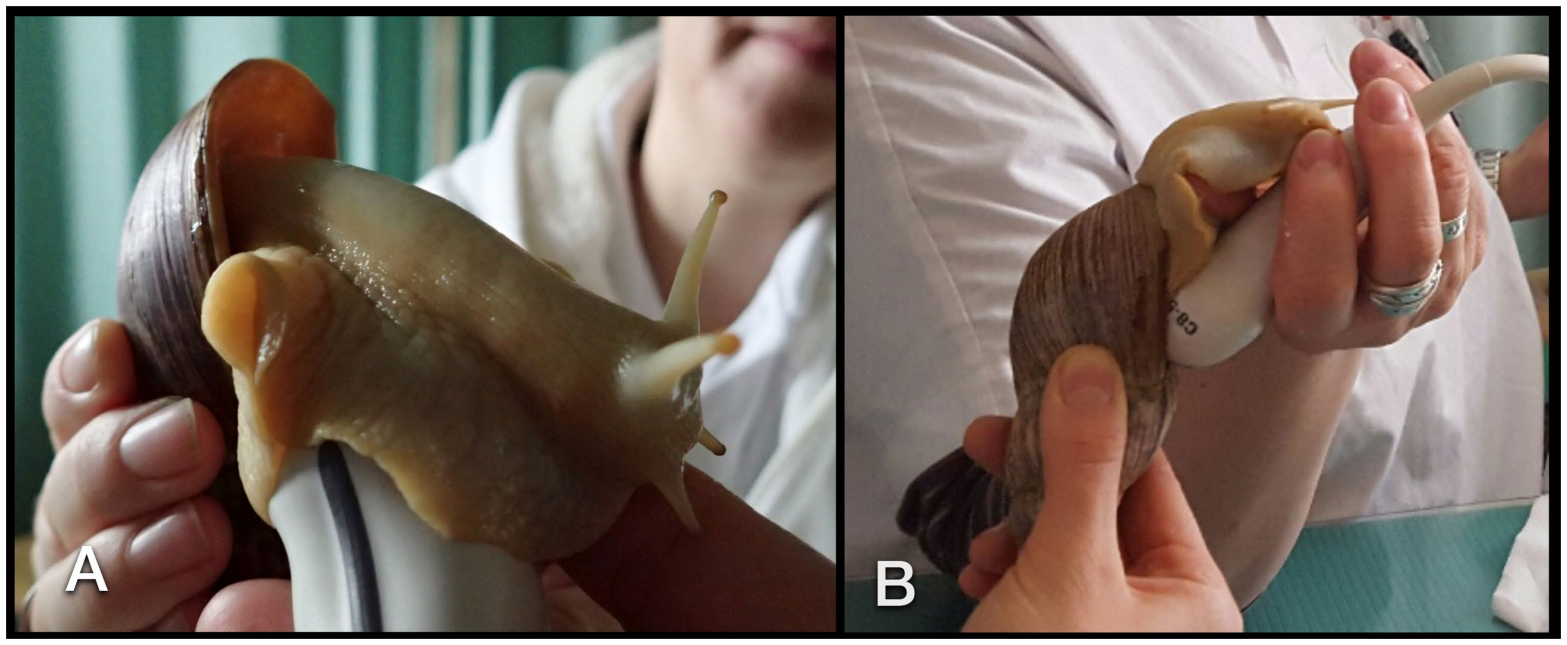
Sonographic examination of a GALS. The animal was soaked to the transducer **(A)**. For visualization of the heart, the transducer had to be angled parallel to the columella **(B)**.

Six sonographic examinations were performed in five conscious GALS. While the animals were held by one person at the shell and foot, a 5–8 MHz micro-convex transducer as well as a high resolution 7–15 MHz linear array hockey-stick probe (iU22 Philips, Bothell, WA) with a small amount of gel or even without any contact medium was attached to the sole of the foot. Usually, the animals began to explore the transducer while creeping over its surface (Figures 4A,B). All animals cooperated because of their curiosity. Obviously, no animal was or could have been forced to participate in these examinations.

During the studies, the snails were kept at a temperature of 20–25°C in a plastic terrarium box equipped with a water bowl, salad, cucumbers, carrots, and dandelions.

All procedures were discussed with and approved by the institutional ethics and animal welfare committee in accordance with GSP guidelines and national legislation (ETK-07109/2015).

### 2.2. Image evaluation

Radiographically, the shape, integrity, and density of the shell were evaluated. We counted the number of whorls. On plain radiographs, the identification, position, shape, size, and density of the lung, pulmonary vein, heart, crop, esophagus, stomach, intestine, kidney, and eggs were noted. In contrast studies of the digestive tract, further evaluation of the location, size, shape, and inner contour of the esophagus, crop, stomach, and bowel loops, including the anus, was possible. The passage time of ingesta was monitored.

The evaluation of the organs in CT studies was similar to that of radiographs; however, the digestive gland (including the sexual segment) and the albumen gland could additionally be differentiated according to their position, shape, size, and density.

Sonographically, the position, shape, echogenicity, inner architecture, and movement (for example, peristalsis) of all identifiable organs were described.

In radiography and CT, the examination procedure always followed the same hierarchy, similar to that recommended for any image interpretation. It was adapted to the snails and was as follows: shell, lung, pulmonary vein, heart, gastrointestinal tract, digestive gland, genital tract, albumen gland, kidney, foot, and muscles. In sonography, the approach and, therefore, accessibility of organs changed continuously due to the animals’ behavior. Therefore, no strict order could be implemented for organ evaluation.

Finally, an anatomic 3D model demonstrating the differentiable inner organs of the snails was created using the software Amira 6.3 (FEI SAS, Mérignac, France; part of Thermo Fisher Scientific™). The shells of the animals serve as landmarks for further orientation, if possible.

No statistics were collected due to the descriptive nature of the study and the low number of participants.

## 3. Results

### 3.1. Imaging procedures

Positioning the GALS on the radiographic cassette for DV views was easily accomplished by placing them on a plexiglass plate. Afterward, the plate was put on a wooden block for the performance of LL and CC views in a horizontal beam. Interestingly, some of the snails turned their head to face the light of the collimator or explored their shadow on the cassette. As manual restraint was not possible, the examiner had to wait for the snail to position itself properly in single cases. Further restraint was not necessary.

**Figure 5:**
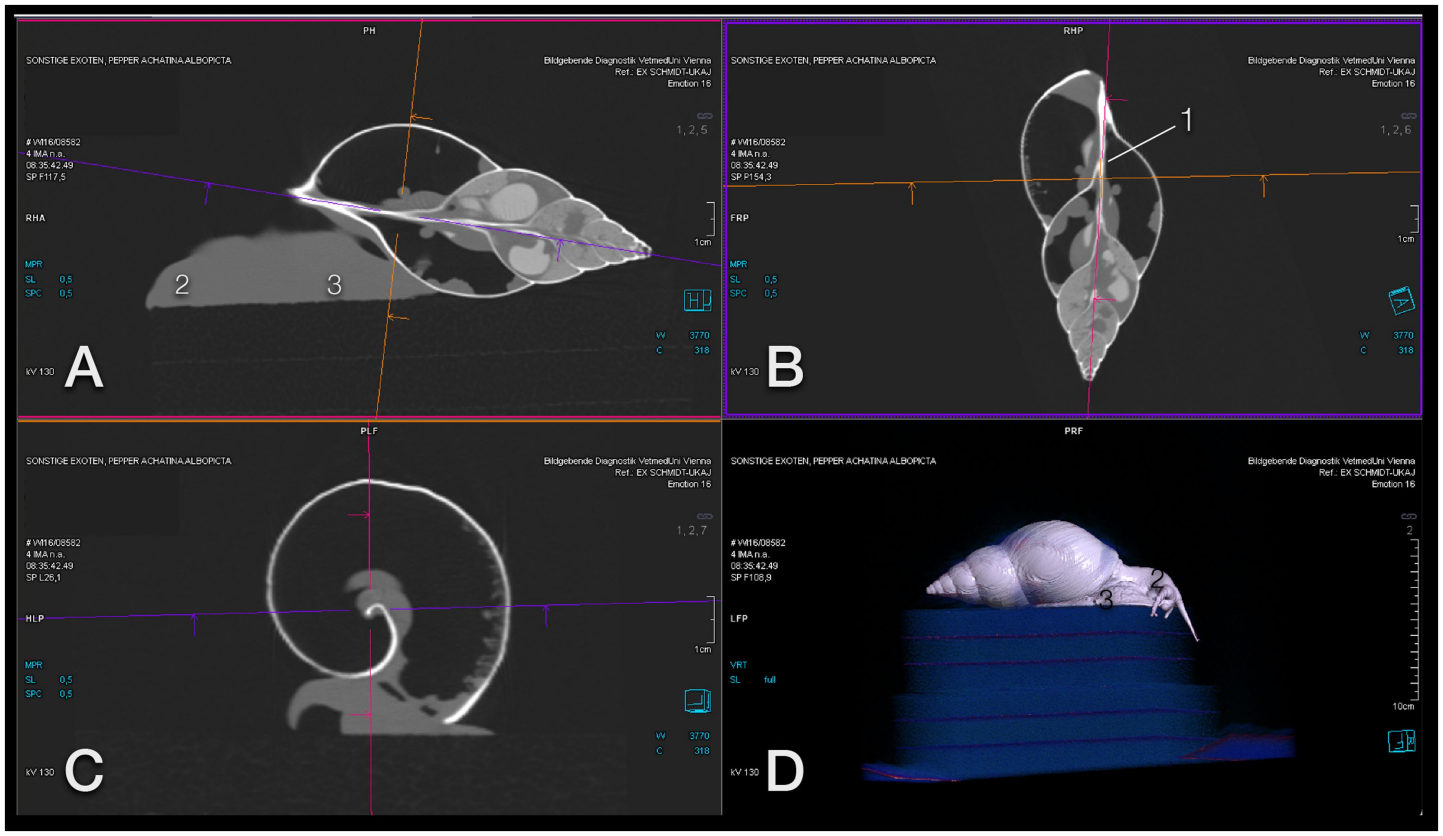
Image orientation for CT interpretation of a GALS. **(A)** Sagittal, **(B)** coronal, and **(C)** transversal plane aligned along the columella (1); the head (2) is orientated to the left **(A)** and top **(B)**, and the foot (3) to the bottom **(A,C)**. This snail was positioned on a foam cube, as is seen on a 3-D model **(D)**, while some others were placed in a plastic box. Note that the animal is exploring the surroundings curiously. Note also that a snail may be quick enough to escape from the scene.

Standard planes had to be declared to be able to evaluate the animals properly in CT examinations. Due to the flexibility of the foot, the shell was the defined reference center (Figures 5A–D). The aperture (shell opening) was directed to the bottom of the image and to the left (for sagittal views) or to the top of the image (coronal view). For all CT examinations, the columella (central axis of the shell) was used for plane orientation. All images were either parallel to the columella (sagittal and coronal views) or perpendicular to it (transverse view). For coronal and transverse views, the right side of the animal was projected to the right side of the image.

It should be noted that snails may move much quicker than expected. Placing them on a block like turtles may not be sufficient. They are also strong enough to lift the lid of a plastic box if it is not secured with tape. Additionally, snails adapt their foot to any provided support. This may result in unexpected, even confusing images (for example, a perfectly rectangular foot, shown in Figure 3D). Some individuals climbed onto their own shells during a scan which resulted in contorted bodies and twisted cranial gastrointestinal tracts (Figure 3B). This was one of the main reasons to use the shell as a landmark for image plane orientation.

After investigating the first snail, the handling of the animals was eased by using the same table position for all of them by painting an outline on the patient table. Therefore, scout views could be neglected, leading to quicker scans. Some animals, which received several CT scans within a few days, seemed to get used to the procedure and stopped exploring the Styrofoam block or box and therefore created hardly any motion artifacts.

All snails appreciated food mixed with contrast medium. The taste or consistency did not irritate them. They fed enthusiastically on vegetables mixed with barium sulfate, and one snail drank the iodine pooling around the contrast-soaked strawberry without any hesitation.

Handling the GALS for sonographic examinations was easy due to the curiosity of most animals. As soon as the transducer was attached to the foot, the animals stuck to it. With slow movements, it was possible to turn the transducer into each desired position. The study was performed with conscious animals. None of them retracted into the shell during sonographic examinations. Therefore, the authors could not assess if there may be an advantage in scanning a withdrawn snail.

### 3.2. Image evaluation

#### 3.2.1. Radiographs

**Figure 6:**
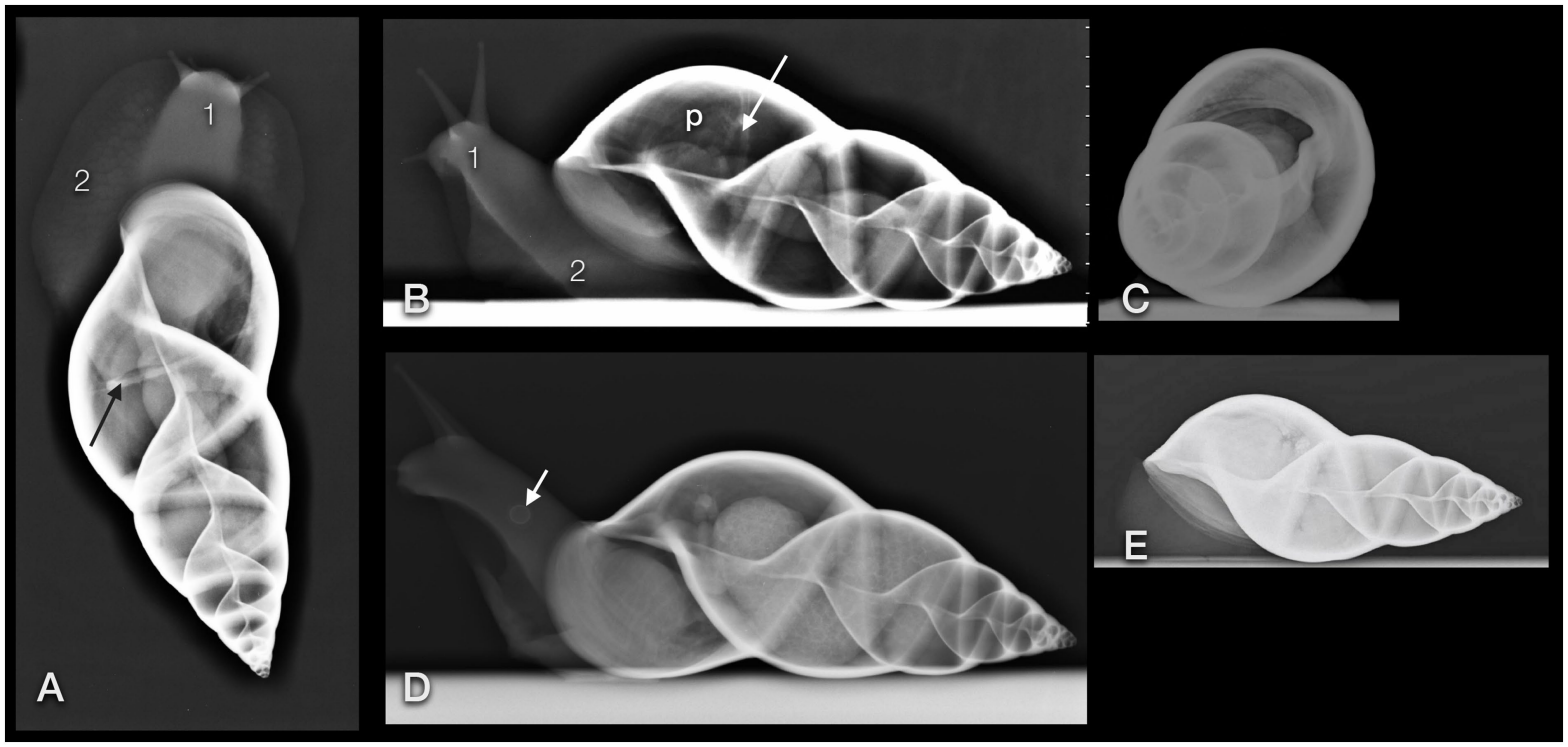
Dorsoventral **(A)**, lateral **(B,D)**, and craniocaudal **(C)** plain radiographs of four GALS (**A,B** are from the same snail). Note that a strict craniocaudal view is often hampered by the downward sloping of the axis of the shell and the position of the snail in general. Note the delicate architecture of the shell. The head (1) and foot (2) of the snail appear as homogeneous soft tissue. Apart from the pneumatic sac (p) and the pulmonary vein (arrow), and in some animals, the kidney or eggs (multiple round thin-shelled structures in **D**), no further inner organs could be clearly differentiated but are represented as tubular shapes surrounding the columella. Differentiation of organs is nearly impossible in snails that are retracted into their shell **(E)**. **(D)** Note the round structure at the foot of the snail (short arrow). It was also visible on the DV view. While the owner suspected a foreign body, the origin could not be found. In a follow-up examination 2 days later, this structure had vanished.

On plain radiographs (Figures 6A–E), the shell presented its unique architecture in bony density with six to nine smooth revolutions and a centrally positioned columella. Usually, the main part of the shell consists of three revolutions, while the others form the tip or spire of the shell. The first whorl is the largest one and includes the apertura.

As the shell whorls are a continuous coil, we defined the first whorl radiographically as follows. When the shell opening was facing ventrally and the tip of the shell to the right, then the first revolution was defined from the most cranial part of the shell (orientated to the left) to a line that runs perpendicular to the columella and through the caudal edge of the shell opening. The differentiation to the second revolution was guaranteed by a deep suture. The part of the shell with the apertura was henceforth addressed as the ventral part and the opposite half as the dorsal part of the shell. The tip of the shell was consequently addressed as the caudal part.

The lung or pulmonary sac was represented by a radiolucent area within the first and second whorl and showed no differentiable inner architecture or border. Therefore, the caudal contour of the lung could not be clearly determined. In some fully relaxed or extracted snails, most of the peripheral lumen of the shell appeared radiolucent and air-filled. The pulmonary vein (also called the efferent branchial vessel in older literature (12)) could be differentiated as thin, soft tissue with a dense linear structure snuggling against the middle to caudal third of the dorsal half of the first whorl.

The heart could be visualized by DV fluoroscopy as a slowly pumping tubular shadow immediately to the right of the columella. In the extracted snail, it was positioned immediately cranial to the suture between the first and second whorl. All other organs appeared as soft tissue density around the columella. The head and foot had the density of soft tissue as well.

**Figure 7:**
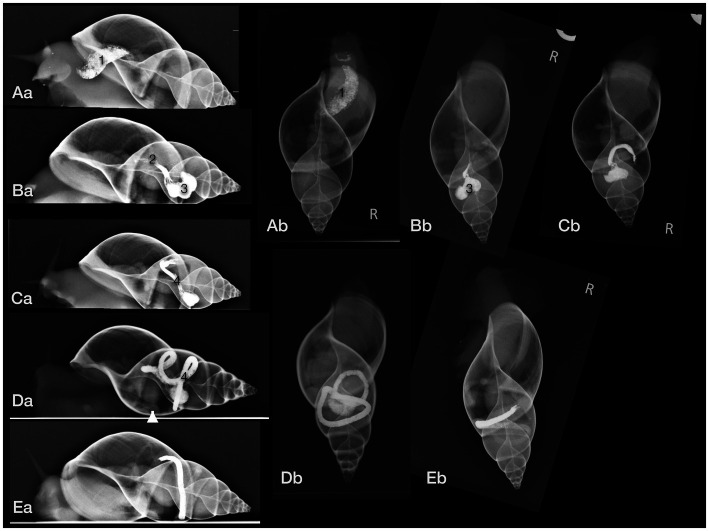
Lateral (a) and dorsoventral (b) radiographs of a GALS after oral barium ingestion. **(A)** The crop (1) was contrastfilled nearly immediately (the radiograph was taken 15 min after feeding began). **(B)** The post-crop esophagus (2) and stomach (3) were contrast-filled after 3 h. **(C)** After 6 h, the stomach and part of the cranial intestines (4) were highlighted, while after 12 h **(D)** most of the contrast medium had proceeded to the intestines. **(E)** After 22 h, nearly all contrast medium had collected in the caudal part of the intestines, cranial to the anus. Note that the shell is always mildly tilted in the different images due to the various postures of the animal. The arrowhead (in Da) points out the kidney.

**Figure 8:**
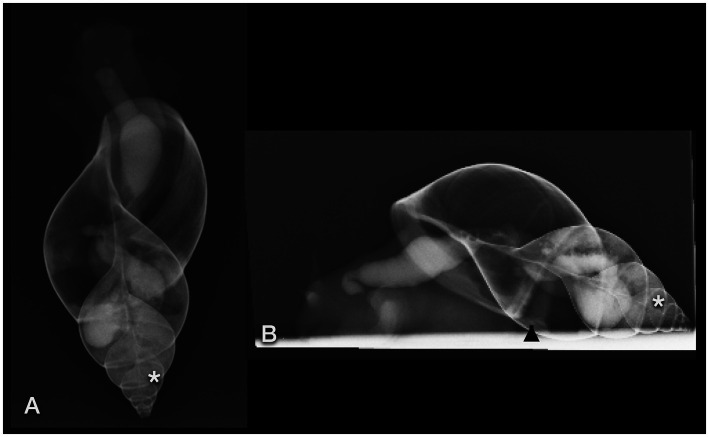
Dorsoventral **(A)** and lateral **(B)** radiographs of a GALS that fed on strawberries soaked in iodine. Note the quite dilated gastrointestinal tract. The contrast enhancement lasted much longer than after barium ingestion. Some iodine was even resorbed to the albumen gland (easier seen in CT) and digestive gland (asterisk), situated within the last three to four whorls. The arrowhead points out the kidney.

The gastrointestinal tract (GIT) could only be differentiated properly after oral contrast medium intake (Figures 7A–E). The radula and mouth were barely enhanced with contrast medium as the food was processed quite quickly. The midsection of the esophagus exhibited a crop. For clear description, the part of the esophagus from the radula and mouth to the crop was addressed as the pre-crop esophagus, and the short part between the crop and stomach was named the post-crop esophagus due to the lack of a neck and thorax in snails. The pre-crop esophagus was highlighted for 1 h in barium sulfate contrast studies, while the crop and post-crop esophagus were marked for up to 5 h. The pre-crop esophagus and the spindle-shaped crop curved mildly to the dorsal within the dorsal half of the foot. In extracted snails, the crop was positioned 50% external to the apertura and 50% within the first half of the first whorl. The post-crop esophagus runs mildly convex dorsally to ventrally before it enters the stomach. The stomach became contrast-filled immediately and emptied after 16 h. It was positioned in the middle of the ventral half of the second to third whorl. The shape of the stomach depended on peristalsis and was more or less u-shaped when visualized on LL views or bilobated to ovoid on DV views. The cranial intestines were contrast-filled after approximately 3 h and up to 25 h and formed a double loop more or less dorsal to the stomach. The simple caudal intestine became enhanced after 10 h and emptied after 36 to 48 h. It was seen as a simple bowel loop running from the left to the right on DV views in the middle of the second whorl and along the suture between the second and third whorl. However, the twist of the snail shell with the continuous helix made it sometimes confusing to point out an exact location. Furthermore, the snails did not always level their shell perfectly horizontally to the radiographic cassette. The anus opened on the right dorsal side of the snail, posterodorsal to the pneumostome (“breathing hole”). When performing an iodine contrast study, the whole digestive tract showed marked contrast enhancement for approximately 13 days. The esophagus and crop appeared wider and more prominent when filled with iodine instead of barium sulfate (Figures 8A,B). An overview of the transit times of all four snails that consumed barium sulfate is given in Table 1.

**Table 1:** GIT contrast medium (barium) passage time observed radiographically in four snails.

Time of contrast	Esophagus	Crop	Crop to stomach	Stomach	Cranial intestines	Caudal intestines
15 min	1	2	2	0	0	0
25 min	1	2	2	0 to 1	0	0
1 h	1	2	2	2	1	0
2 h	0	2	2	2	1	0
3 h	0	2	2	1 to 2	0 to 1	0
4 h	0	2	2	2	2	0
5 h	0	0 to 1	1	2	1 to 2	0
10 h	0	0	1	2	2	0 to 1
11,5 h	0	0	0 to 1	2	2	1
13 h	0	0	0 to 1	2	2	2
14,5 h	0	0	0 to 1	2	2	2
16 h	0	0	0 to 1	2	2	2
21 h	0	0	0 to 1	0 to 1	2	2
22 h	0	0	0 to 1	0 to 1	1 to 2	2
24 h	0	0	0	0 to 1	2	1
25 h	0	0	0	0	2	2
33 h	0	0	0	0	0	1
36 h	0	0	0	0	0	1
48 h	0	0	0	0	0	0

Subjective evaluation of the amount of visible contrast medium was graded as 0 – no contrast medium present, 1 – a little contrast medium present, and 2 – large amounts of contrast medium present.

Neither the albumen gland nor the digestive gland could be clearly differentiated on plain radiographs. However, the digestive gland could presumptively be identified due to its typical location within the tip of the spire. These glands were assumed to be represented by the dense opacifications of soft tissue between the contrast-filled GIT. On the other hand, the digestive gland and the albumen gland were enhanced after oral iodine ingestion and could be visualized as mildly opaque structures (Figures 8A,B).

A clutch of eggs was visible in one snail (Figure 6D), while no other part of the reproductive tract could be seen on any radiograph. Unfortunately, no sonographic or CT examination took place on this pregnant GALS before the clutch was delivered.

The single kidney or so-called nephridium was a flattened, tongue-shaped organ that was attached to the inner contour of the ventral part of the first whorl, immediately caudal to the apertura and more or less at the level of the centrally located heart (Figures 7D 8B). It reached to dorsal bilaterally. When viewed from the cranial position on a CC view, it was positioned from half past four to nine o’clock.

The feet of the snails appeared as soft tissue, as did the columellar muscle.

#### 3.2.2. Computed tomography

**Figure 9:**
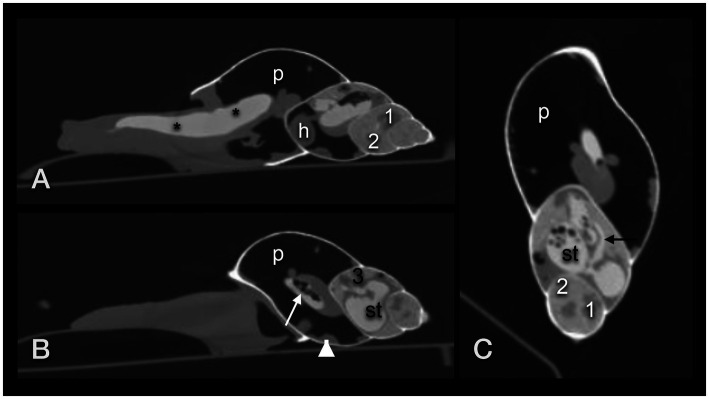
CT in the modified bony window of a GALS 15 min after ingestion of strawberries soaked with iodine contrast medium. Sagittal plane mildly right paramedian **(A)** and in the right lateral half of the shell **(B)**, and coronal plane **(C)**. Note that iodine seemingly causes a widening of the pre-crop esophagus and crop (asterisk), so that the connection between the two is poorly demarcated. The white arrow in **(B)** points out the post-crop esophagus. The black arrow in **(C)** points out the connective duct between stomach and albumen gland. h – heart, p – pneumatic sac, st – stomach, 1 – sexual segment, 2 – digestive gland, 3 – albumen gland, arrowhead – kidney.

**Figure 10:**
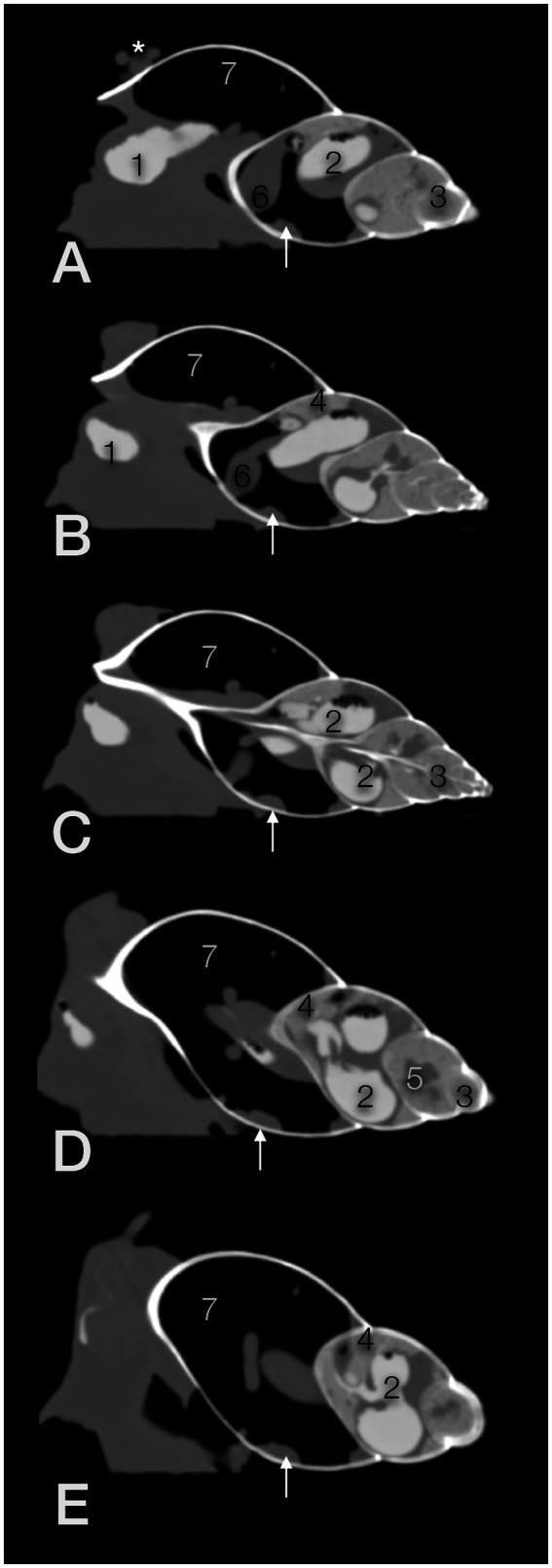
Exemplary sagittal CT images (in a modified bony window) from the right **(A,B)** to the left side (**D,E; C**; in the center) of a GALS. The head is to the left. The animal was fed on strawberries with an iodine contrast medium 3 h prior to the scan. The soft tissue structures dorsal to the shell in (A, asterisk) belong to the head and foot of the snail. 1 – crop, 2 – u-shaped stomach, 3 – digestive gland, 4 – albumen gland, 5 – sexual segment, 6 – heart, 7 – pulmonary sac (capillary network not seen in these window settings), arrow - kidney; compare Figure 11.

**Figure 11:**
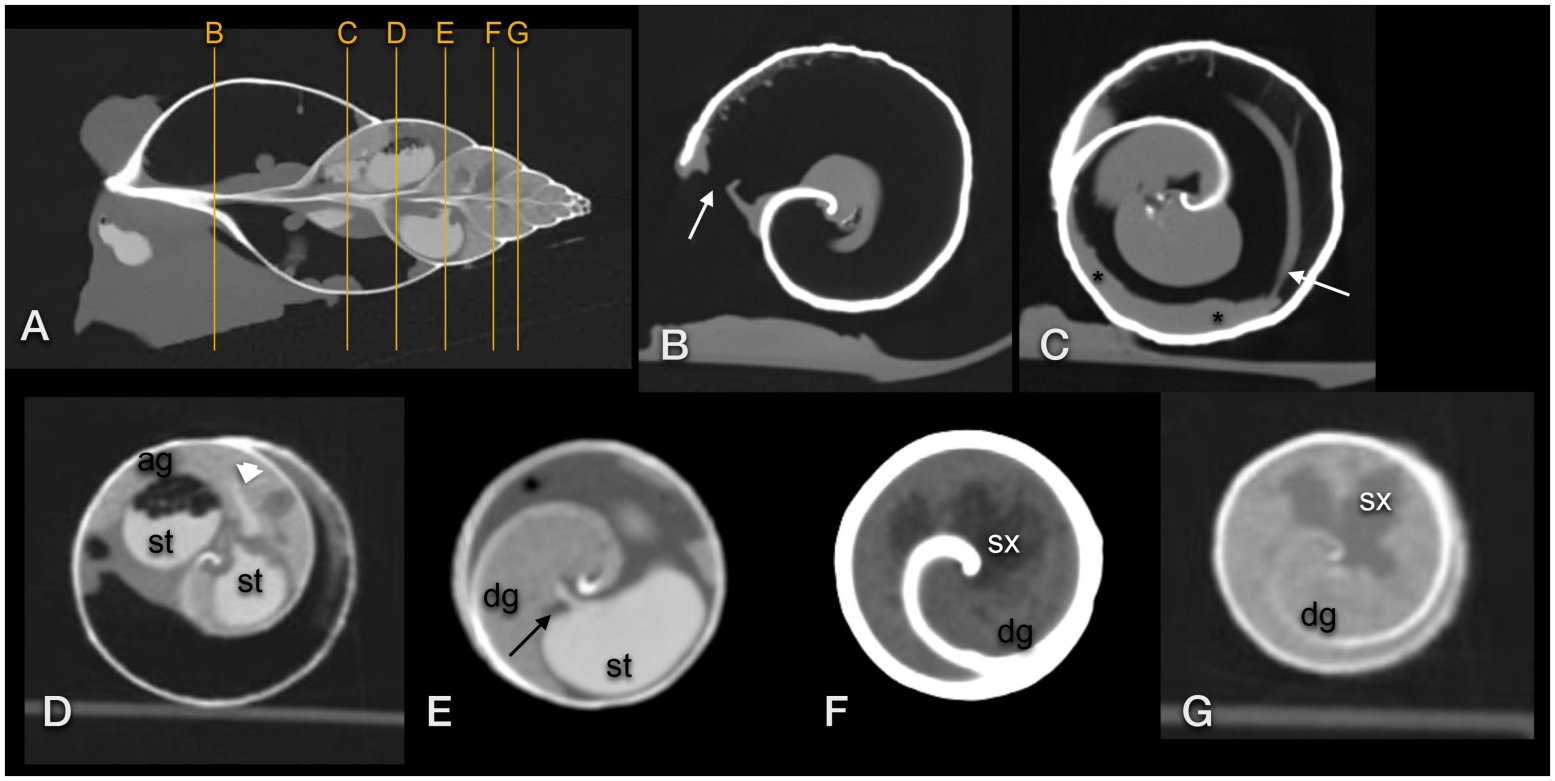
Transversal CT of a GALS (same animal as in Figure 10): **(A)** sagittal image used as topogram, **(B–D,G)** in a modified bony window, **(E,F)** in modified soft tissue windows. **(B)** demonstrates the pneumostome (arrow) and simple lung (large air space). A reticular net of tiny vessels was present **(B,C)**. **(C)** The kidney (asterisk) was positioned along the wall of the first whorl and was surrounded by the lung. The efferent branchial vessel or pulmonary vein was in connection with the kidney (arrow). **(D)** The stomach (st) was surrounded by the albumen gland (ag). A thin contrast-enhanced duct (arrowhead) connected the stomach and gland. **(E)** A thin contrast-enhanced duct (arrow) emptied from the digestive gland (dg) into the contrast-filled stomach (st). **(F,G)** The lobulated sexual segment (sx) was hypodense in comparison to the hyperdense digestive gland (dg).

**Figure 12:**
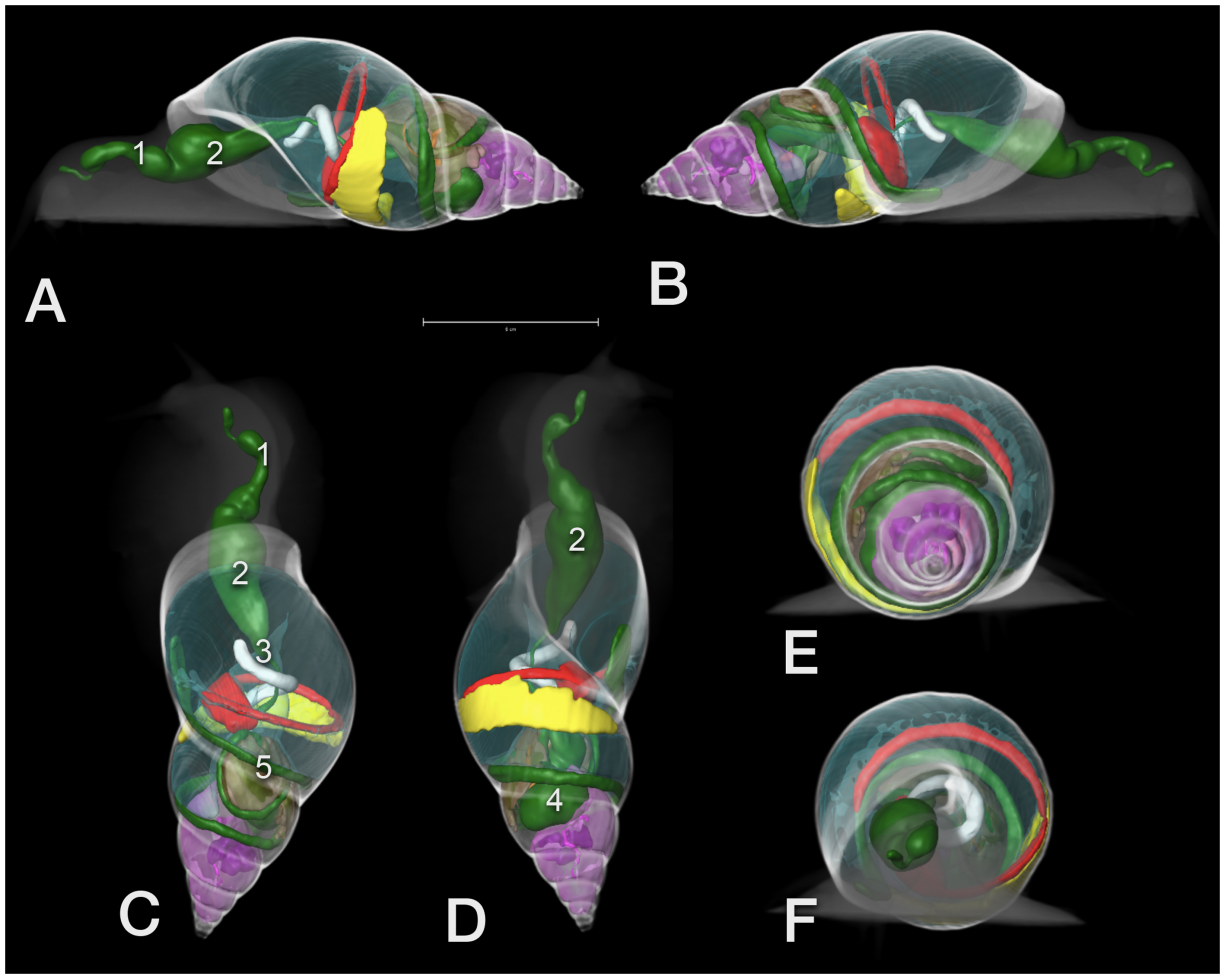
3D model of the CT of a GALS. **(A)** View from left lateral, head to the left, **(B)** from right lateral, **(C)** from dorsal, **(D)** from ventral, head to the top, and **(E)** view from the tip of the shell (caudocranial) as well as **(F)** from cranial. The scale in A indicates a 5 cm length. Note the clear demarcation of the different whorls. Green color: GIT: 1 – pre-crop esophagus, 2 – crop, 3 – post-crop esophagus, 4 – stomach, 5 – bowel loops, red: heart and main vessel, brown: albumen gland, pink: digestive gland (with the embedded purple sexual segment), yellow: kidney, transparent blue: pneumatic sac; the head and shell are shown like a radiograph in different shades of grey.

Unsurprisingly, the delicate architecture of the calcified shell was visualized perfectly with CT (Figures 9–12). The image orientation was the same as described above. In CT and on radiographs, the wall of the shell seemed to be single layered.

The lung was situated in the first whorl (Figures 9A–C, 10). A kind of netlike or honeycomb-like ”parenchyma”, representing a plexus of pulmonary vessels, covered the inner contour of the dorsal half of the palatal wall (outer wall of the shell). This plexus was accompanied by the large pulmonary vein that finally approached the nephridium (Figure 11C). In some animals, tiny mineralized particles were found within this plexus. The pneumostome (Figure 11B) could be easily seen craniodorsally on the right side of the mantle. The heart was a quite slim oval structure that was easier identified after performing the fluoroscopic examination (Figures 10 12).

The foot showed a hypodense center (220 to 230HU) and a mildly hyperdense peripheral rim or contour (270 to 300HU), easily seen on a modified soft tissue window. The mouth and radula were hyperdense as well. In the dorsal half of the foot, elongated hyperdense structures represented the pre-crop esophagus and the crop. They could be identified more easily when filled with gas bubbles or mineralized food particles. The post-crop esophagus and stomach were presumptively differentiated in extracted snails, while the bowel loops were only seen when filled with ingesta (Figures 9 10). After the ingestion of the contrast medium, the gastrointestinal tract was highlighted excellently. The iodine drink, in particular, caused a very homogeneous filling of the quite extended organs. An adapted lung window instead of a soft tissue window was preferred for image evaluation of contrast-enhanced images.

The albumen gland was the densest soft tissue organ within the shell, measuring up to 400HU, and was situated dorsally in the second whorl (Figures 9–11). It tended to reach a little bit more to the right than to the left. In larger individuals, it measured an estimated 63 mm in length (given its semicircular shape) and 12 mm in width. The digestive gland (liver) occupied the third to seventh or eighth whorl and showed a left or anterior lobe that reached from ventral to median while traversing the second whorl (Figures 9–11). The tip of that lobe even reached the first whorl, adjacent to the intestines and nephridium. The right or posterior lobe was more coiled and occupied the top of the spire. In the left half of the third to fourth whorl, a cauliflower-like, hypodense area (250HU) was embedded in the hyperdense digestive gland (290 to 325HU), representing the sexual segment or ovotestis (Figures 9–11). The digestive gland was denser than the intestines but less dense than the albumen gland. Both glands were homogeneous but contained some prominent hypodense kind of ducts.

The penis appeared as a hyperdense structure caudal to the eyes at the right side of the head. In close proximity, the genital antrum could be localized, mostly because it contained a tiny amount of air. Other structures of the reproductive tract could not be clearly differentiated in CT.

The nephridium was proven to be situated along the inner contour of the outer wall of the first whorl of the shell, as described on radiographs (Figures 9–11). Its surface seemed to be very mildly bumpy, and the end slightly blunted. It was somewhat hypodense in comparison to the other viscera but, in general, of soft tissue density (20 to 120HU).

As soon as nearly the whole digestive tract was highlighted with contrast medium, 3D models were created for demonstration of the anatomy. The shell was used as a reference model (Figure 12).

#### 3.2.3. Sonography

**Figure 13:**
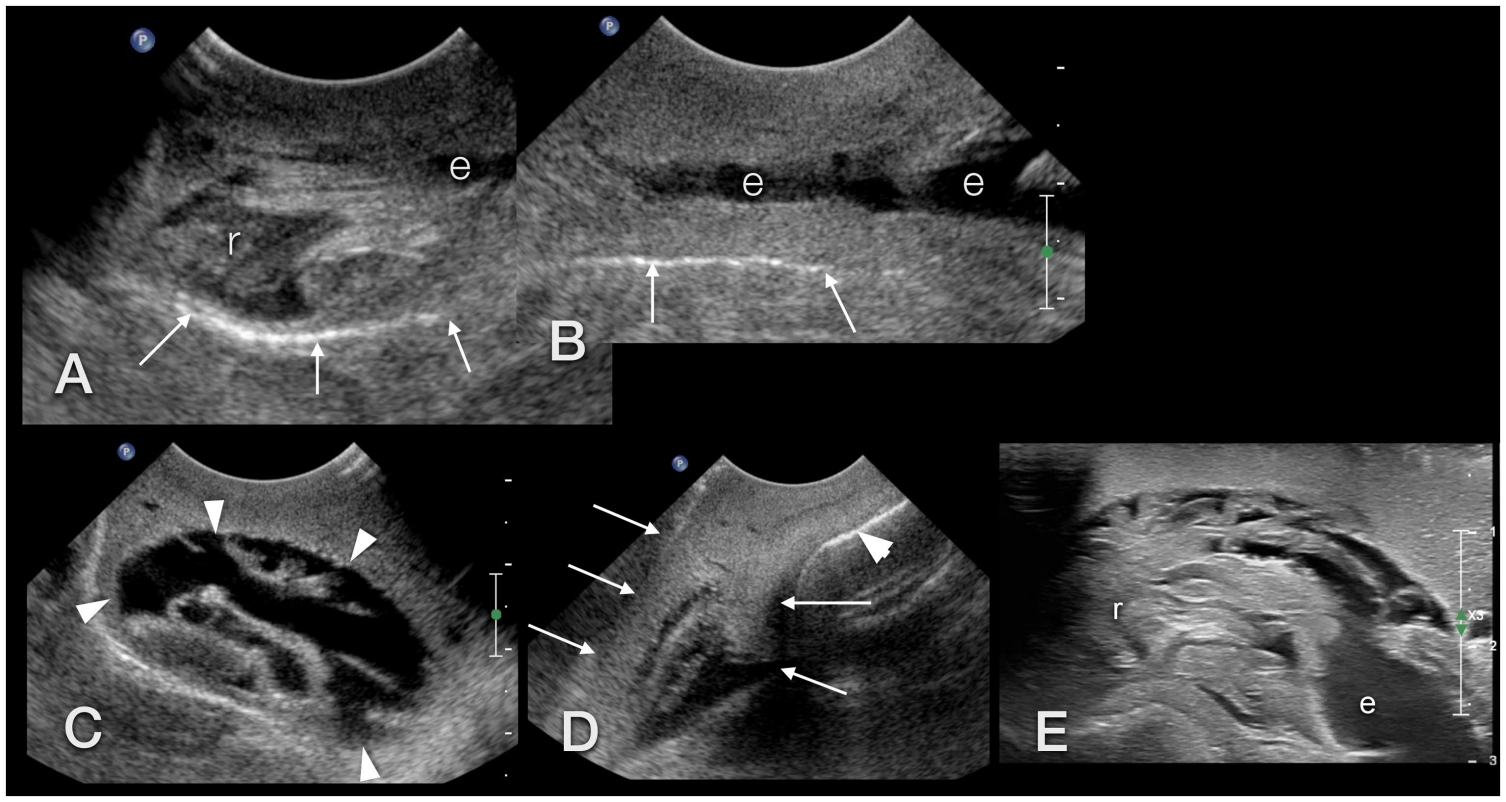
Sonographic examination, with a micro-convex transducer **(A–D)** and with a high-resolution linear hockey-stick transducer **(E)**, of the upper gastrointestinal tract in a GALS, sagittal plane, head to the left. **(A,B)** represent a kind of a panorama view of the whole pre-crop esophagus. The outer contour of the animal is marked with arrows. The radula (r) was easily identified by its symmetric movement **(A,E)**. The pre-crop esophagus (e) was fluid filled in all examined GALS **(B)**. The crop (arrowheads) had an oval shape and various contents (here: fluid), depending on the food of the animal **(C)**. The foot (arrows) of the GALS appeared hyperechoic, similar to a fatty liver in a dog or reptile. The shell (arrowhead) hindered further examination of the inner organs **(D)**. **(E)** While the busy radula and cranial part of the fluid-filled esophagus could be identified with reasonable certainty, many structures were seen that could not be doubtlessly identified. Therefore the buccal mass may have been seen but was not identified clearly.

**Figure 14:**
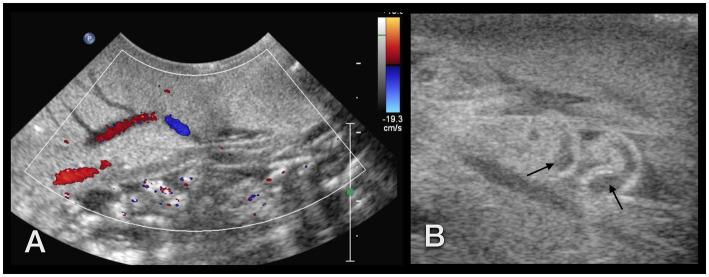
**(A)** Sonographic examination of the sole of a GALS. Using Doppler sonography helped to differentiate vessels. **(B)** Right lateral of the head of a GALS. A tubular, tightly packed hypoechoic structure with hyperechoic walls was interpreted as the cranial part of the penis (arrows), as it could be followed to the common genital opening. With Doppler sonography, it could be clearly differentiated from nearby vessels. Note the use of two different transducers and different resolutions: in **(A)**, a 5–8 MHz microconvex transducer, and in **(B)**, a 7–15 MHz hockey-stick linear transducer was used.

The foot, radula, esophagus, crop, heart, and part of the genital tract (penis, genital antrum) were visualized sonographically. The foot was homogeneous, finely granulated, and hyperechoic: similar to a dog suffering from hepatic lipidosis (Figures 13A,B,D). Some vessels demonstrating clear fluid movement using Doppler sonography could be identified within it (Figure 14A).

The simple tubular heart, built of a single auricle and a single ventricle, could be reached by orienting a transducer nearly parallel to the columella (Figure 4B). It could be clearly identified due to its rhythmic pumping contractions. Although Doppler sonography was tried, it was not very conclusive to the authors. The direction of flow could be detected, but further details could not be differentiated.

The radula appeared as a kind of bilobated lip of moderate echogenicity showing symmetric movement (Figure 13A). The esophagus was, in all animals, fluid-filled and occupied a fifth of the diameter of the foot of the snail (Figures 13A,B). A very thin, hyperechoic line or wall bordered the esophagus. The crop appeared as an ampule and was bordered similarly to the esophagus. Depending on the feeding status, it was filled mostly with fluid and some hyperechoic contents. It could be followed through the mantle and apertura into the shell, but its entrance into the stomach could not be reached (Figure 13C). All other parts of the gastrointestinal tract, as well as the digestive and albumen gland, could not be visualized. An approach through the shell failed due to its mineralization.

On the right-hand side of the head of the snail, caudal to the eyes, at the level of the common genial opening, the cranial part of the penis was seen as a small, seemingly tubular, tightly packed hypoechoic structure with hyperechoic walls of nearly the same echogenicity as the foot (Figure 14B).

The caudal part of the penis, vas deferens, free oviduct, uterus, and flagellum should be positioned immediately dorsal to the foot but could not be visualized sonographically. The ovary and testis are located deep within the shell and were, therefore, not accessible as well.

## 4. Discussion

### 4.1. Technique

The GALS were very curious and cooperated surprisingly well in all imaging procedures. Sometimes one individual retracted into the shell when being exposed to the collimation light for radiography but soon after bulged its eyes to spot its shadow on the cassette. During the sonography procedure, they explored the transducer and did not retract when confronted with ultrasound gel. This is contrary to the report of Pizzi (19), who preferred using only water as a coupling medium. Additionally, the snails were not as slimy as one may expect. The authors sometimes underestimated the velocity and agility of the participants. Radiographs and CT examinations had to be performed quickly to avoid motion artifacts, especially in CT. However, anesthesia was not needed for any examination.

The two different contrast media were offered via favored food. Because force-feeding is barely possible in snails, the authors relied on the cooperation of the animals. However, we were still surprised at how enthusiastically they fed on strawberries soaked in iodine or cucumbers in barium. Frye described this fondness for strawberries in 1992 when performing one of the first radiographs of a slug with gastrointestinal contrast medium (20).

### 4.2. Anatomy and image interpretation

Due to the complex anatomy of GALS, the authors decided to project the right side of the animal to the right side of the image to minimize further confusion, although it is an international standard agreement to project the right side of the patient to the left side of the image in diagnostic imaging.

The body of GALS consists of four main parts: The shell, the head, the foot, and the visceral sac. The shells of our snails were dextral, as in most GALS. The first revolution—equaling nearly half of the length of the shell (2)—is named the body whorl because most of the visceral mass of the animal resides here. The remaining whorls are collectively known as the spire (32). Usually, the whorls of a snail’s shell are counted from the apex to the aperture (main shell opening). However, the first whorl of that pointed spire may be very small and, therefore, sometimes difficult to differentiate on radiographs. On the other hand, some authors count the body whorl as the first whorl (33). For practical purposes, the authors of the present study labeled the largest whorl with the apertura as the first whorl or body whorl. It is separated from the second whorl by a deep suture. Cooper (10) mentioned three layers of the shell: an outer, colored horny periostracum, the middle ostraceum, which is formed by calcium carbonate, and an inner layer that is secreted by the mantle. In radiography and CT, only a single mineralized wall or layer could be seen, while the architecture, in general, could be visualized perfectly. However, the shell was thick enough to block sonography.

The internal anatomy of gastropods is determined by their development, which includes a 180-degree torsion of the body (33, 34). The lung is represented by the pulmonary sac, a transformed part of the mantle. It opens via the contractible pneumostome at the right-hand side of the neck of the snail (2). Along the inner contour of this sac, netlike structures, representing a plexus of pulmonary vessels, can be found. All these structures could be easily seen in CT, while on radiographs, only the lucent area of the lung could be depicted. Some mineralized particles within the air chambers between the plexus were interpreted as foreign material that could have entered the lung via the pneumostome.

The two-chambered heart has a thin-walled, wine-glass-shaped auricle that is connected to the pulmonary vein and a single ventricle with a thicker, spongy wall within the thick pericardial sac (10). It beats 25–60 times per minute. The anterior part of the heart is orientated parallel to the nephridium. The pericardium is in direct communication with the lumen of the kidney, which is, therefore, constantly fluid-filled. Oxygenated hemolymph from the respiratory plexus runs through an efferent branchial vessel through the auricle (pulmo-auricular orifice) and the muscular ventricle into the aorta, the anterior cephalic artery, and the posterior visceral artery. Thus, the heart carries only oxygenated arterial blood (2). Blood collects in visceral sinuses as no connection between arterial and venous capillaries exists (a so-called open circle). An afferent branchial vessel finally empties into the respiratory plexus (2, 11).

In the present study, the heart was best evaluated with a combination of fluoroscopy and sonography. It was easy and beneficial to attach the snails to a perpendicular surface to achieve perfectly aligned fluoroscopy videos of the heart. Position, size, cardiac action, and valve motion were seen as soon as an appropriate plane was implemented radiographically and sonographically. No contrast-enhanced angiography, cardiac ventriculography, or nephrography was performed. Alternatively, an intramuscular injection of iodinated contrast medium could be tried, but it was not part of the present study and not part of the application to the ethics and animal welfare committee. This would be an object for another imaging study. However, in the literature, the injection of contrast medium into the coelomic cavity is listed, but unfortunately, neither the exact procedure nor the results, advantages, or disadvantages are described in any way (18). Nevertheless, the authors could clearly identify the heart in plain fluoroscopy and sonography. It was stated that the heart is situated deep within the cavity of the shell and, therefore, inaccessible sonographically (35). Therefore, the cephalic artery was used to monitor cardiac activity. Conversely, the authors of the present study could reach the heart when angling the transducer nearly parallel to the columella at quite a flat angle (compare Figure 4B). The larger vessels could be followed cranially and to the foot. However, no attempt was made to identify the single branches.

According to the literature, the simple tubular digestive system of this terrestrial species begins with a mouth and ends with an anus. The different sections are the radula (for rasping food), the buccal cavity supported by the so-called buccal mass, a thick-walled esophagus, a thin-walled crop, the stomach, a thin-walled intestine, the rectum, and the anus (2, 11, 33). The crop lies within the ultimate whorl of the shell and is used to store food and initiate digestion. The paired salivary glands lie at the caudal part of the esophagus, adherent to its dorsoanterior walls (2, 10). Food passes from the crop into the simple stomach and then from the left side of the stomach into the intestinal canal, which passes through the tissues of the digestive gland. The junction of the esophagus and crop is marked by the larger lumen of the latter (11). The stomach lies embedded in the digestive gland and is more or less heart-shaped (2). The intestines arise from the left anterior border of the stomach, create nearly a “U” shape around the crop, form an arch, proceed along the posterior end of the kidney, turn to the right and dorsal, and embed in the mass of the digestive gland (11). Finally, they run posterolaterally at the right border of the visceral mass, form another loop, and end in the rectum and anus, the latter being situated behind the breathing hole at the right side (10). Our contrast studies confirmed this description but could not identify the salivary glands, nor could sonography. Otherwise, sonography was able to observe the movement of the radula during feeding and the peristalsis of the esophagus and crop.

The literature moreover describes the bi-parted crop entering the stomach as a short, broad duct directly from the right side (11). The authors of the present study found that, in contrast studies, the narrow tubular structure between the sac-like crop and the stomach resembled much more the appearance of the esophagus than the second part of the crop described in the literature, therefore being similar to avians. Usually, the different parts of the esophagus are addressed in mammals as the cervical and thoracic esophagus. Because GALS lack a true neck or even thorax, the authors suggested identifying a pre-crop and post-crop esophagus in terms of diagnostic imaging, while in histology, this “post-crop esophagus” was identified as part of the crop (11).

Although the authors provide the gastrointestinal passage time for the presented four snails, the number of individuals is too low to draw general conclusions about passage times in GALS. It can be assumed that it may vary with season and temperature, similarly to reptiles, as both are ectothermic (36).

The digestive gland, or “liver,” a reddish-brown organ, consists of two parts (10). The larger left lobe lies anterior to and is traversed by the intestine. The twisted right part (posterior) occupies the uppermost coils of the shell (37). This gland secretes digestive enzymes into the stomach. The hepatic cells metabolize the absorbed nutriment and excrete waste into the intestine. Furthermore, some cells serve as calcium reservoirs for shell formation. Due to this hidden position, we could not approach the digestive gland or most of the GIT sonographically in the present study.

On plain radiographs, the confluent soft tissue opacification of all viscera could not be differentiated further. With oral contrast medium studies, the gastrointestinal tract was highlighted. It could therefore be assumed that the other soft tissue structures originate from the digestive and albumen gland as well as gonads and muscles. However, proper serosal detail was lacking radiographically.

The single (usually left, as the right one has disappeared during evolution) nephridium or kidney of *Lissachatina fulica* is positioned transversely-oriented parallel to the heart (2) and is described as a single-lobed, thin structure with a compressed lumen much flattened in one dimension (10, 37, 38). The average size in a full-grown snail is approximately 5×1.2 cm. The internal surface is greatly increased by the presence of folds running across the lumen. Efferent hemolymph drains via the respiratory plexus into the efferent pulmonary vessel. A split ureter ends into the front, more rounded end of the kidney (37). The posterior part lies close to the intestines.

We could not visualize the seemingly fluid-filled lumen and the above-described internal folds. On radiographs, the kidney appeared of homogeneous soft tissue density, while it was more heterogeneous in CT. It seems that due to beam hardening artifacts caused by the shell and unevenly distributed inner organs, the peripherally positioned nephridium showed a wide range of densities. Sonographically, the internal structure of the kidney should be visible, but in living animals, it cannot be accessed due to its peripheral position in the shell and the surrounding lung. The reno-pericardial canal, which should connect the pericardium with the lumen of the kidney, could not be visualized either (38). For proper visualization of such a canal, an intramuscular iodine contrast study may have been useful but was not performed as already mentioned.

The reproductive system in these hermaphrodites comprises the ovotestis, ovotestic duct, uterus, sulcus or sperm groove, prostatic acini, albumen gland, efferent ducts, oviduct, vas deferens, spermatheca, vagina, and penis, as well as the genital aperture (12, 15). The genital aperture and penis could be identified at the level of the head with CT and sonography. The lobulated ovotestis (or hermaphrodite gland) is lodged in the posterior part of the digestive gland towards the inner border of the spiral. It could be differentiated from the digestive gland due to its reduced density already on plain CT images in the present study. The digestive gland was clearly seen on plain and contrast-enhanced CT images in any case.

The albumen gland is situated anterolaterally more or less parallel to the proximal rectum, dorsal to the ovotestis duct and ovisperm vesicle, and ventral to the digestive gland. It is a lighter brown than the dark brown digestive gland or even a whitish color (2). It is a part of the female reproductive tract, contains a fertilization chamber for the eggs, secretes perivitelline fluid, and serves as a reservoir for many different minerals (37). Its size is highly correlated with the weight of the snail and its reproductive status and can measure up to 2.03 (±0.15) cm in length and 0.51 (±0.09) cm in width (5). The albumen gland enlarges during the breeding season up to 6.2 × 1.8 cm (12). *A. marginata* is known to have the largest albumen gland, which may correlate to their large eggs with thick shells (5). The different macroscopic appearance, as well as the different functions, may be responsible for the different densities of the albumen and digestive gland in CT. Although the albumen gland was easily depicted in CT images, measuring the dimensions was tricky due to the semicircular shape. However, the dimensions were similar to those observed by Ghose (12) and may therefore indicate snails in a breeding condition.

Surprisingly, the vagina, uterus, and the various ducts could not be seen at all. The structures located dorsal to the foot, in particular, were expected to be visualized with sonography, but only a small part of the penis could be differentiated at the right-hand side of the head. It may be that the other genital structures are too delicate to be differentiated. On the other hand, the prostate gland is a multi-folded, quite large structure that was expected to be seen—but was not. Magnetic resonance imaging (MRI) is prone to show soft tissue structures and could, therefore, theoretically be the imaging modality of choice to evaluate those parts of the genital tract.

The columellar muscle complex connects the snail to its shell. It is divided into two parts: the pedal retractor and the free retractor muscles (2). They are attached to the columella, close to the junction of the body and penultimate (or second) whorl. The pedal retractor muscle spirals around the columella and anchors the snail in the shell. The free retractor muscle is proximally attached to the columella beyond the origin of the pedal retractor muscle. It divides into several branches and subbranches to support and retract the tentacles, buccal mass, snout, vas deferens, and anterior part of the foot (2). In CT and radiography, the columellar muscle appeared as a string of soft tissue, but the single strands were not identified in the present study.

One main intention of the study was to provide the imaging anatomy of GALS and to compare the various imaging techniques. Surprisingly, sonography was less valuable than expected in these mollusks. Nevertheless, as the authors became more familiar with the anatomy of GALS, more and more details could be detected, even on plain radiographs. Differentiation of the organs was easier in extracted snails than in retracted ones. Cardiac action could be observed sonographically and in fluoroscopy. The kidney could be seen on plain radiographs as well as in CT but could not be approached sonographically. In conclusion, CT after oral contrast uptake was the most valuable imaging tool.

Obviously, micro CT offers the most detailed visualization of all organs in snails but is usually performed in stained dead specimens and, therefore, not appropriate for patients (22, 23). Although CT would be the modality of choice for GALS, CT examinations are quite cost-intensive, which may limit owner acceptance. However, the information gained in this study can be transferred to radiography. Renal enlargement, in particular, is a roentgen sign of renal disease that could be diagnosed in patients (39).

A limitation of this descriptive study may be that only six snails were used in this study. On the other hand, one may assume that snails of the same family should have similar, if not the same, anatomy (14). The animals belonged to different owners. Therefore, they are most likely not related to each other, which confirms the regularity of the above-demonstrated anatomy.

A complementary imaging technique to CT would have been MRI. Mollusks represent ideal candidates for MRI, and this procedure has proven to be the most useful non-invasive technique for gastropods in research but was not part of the present study (31). The main reason was that the authors wanted to achieve information for the practitioner as quickly as possible, and MRI is not as easily available as radiography, sonography, or CT. Furthermore, MRI is more expensive and much more time-consuming. However, MRI has been used to evaluate the sexual maturity of living endangered limpets (40). The size of the gonads and the digestive gland was a relevant criterion. The digestive gland could be seen clearly using plain CT in our studies.

As a personal, not purely scientific observation, the authors would like to mention the variable characters of the participants of our study. It was very interesting to follow the different behavior of the snails during the various imaging procedures. While one was quite shy, another one was overwhelmingly curious, exploring its shadow on the radiographic cassette or the sonographic transducer. Those people who invest time and passion in interacting with these snails will easily understand why individuals in kindergartens or schools cannot be simply replaced by another snail. Owners grow very fond of them, although interaction with them is definitely different than with mammals. Therefore, the authors want to encourage further diagnostic procedures and treatment in these mollusks.

## Data availability statement

The original contributions presented in the study are included in the article/supplementary material, further inquiries can be directed to the corresponding author.

## Ethics statement

All procedures were discussed and approved by the institutional ethics and animal welfare committee in accordance with GSP guidelines and national legislation (ETK-07109/2015).

## Author contributions

MG and SS-U contributed to the conception, design of the study, and wrote sections of the manuscript. MG performed all imaging studies and wrote the first draft of the manuscript. Interpretation of the imaging studies was performed in consensus between MG and SS-U. SH reconstructed the colored 3D models. All authors contributed to the article and approved the submitted version.

## Abbreviations

ag: Albumen gland
CC: Craniocaudal
CT: Computed tomography
dg: Digestive gland
DV: Dorsoventral
f: Foot of snail
GALS: Giant African land snail
h: Heart
HU: Hounsfield Units
LL: Laterolateral
p: Pneumatic sac
st: Stomach
sx: Sexual segment

## References

[1] Proios K Cameron RA Triantis KA. Land snails on islands: building a global inventory. Front Biogeography. (2021) 13:51126. doi: 10.21425/F5FBG51126

[2] Srivastava PD. Problem of land snail pests in agriculture. A study of the Giant African snail. New Delhi, India: Ashok Kumar Mittal, Concept Publishing Company (1992).

[3] Henderson I. Slugs and snails in world agriculture (BCPC monograph no. 41). British Crop Protection Council: United Kingdom (1989).

[4] Albuquerque FP-A Assunção-Albuquerque MJT. Distribution, feeding behavior and control strategies of the exotic land snail Achatina fulica (Gastropoda: Pulmonata) in the northeast of Brazil. Braz J Biol. (2008) 68:837–42. doi: 10.1590/S1519-69842008000400020, PMID: 19197503

[5] Ademolu KO Akanmu ET Dedeke GA Jayeola OA. A preliminary chemical and structural analysis on the albumen gland of three snail species found in Abeokuta, Ogun state, Nigeria. J Trop Agric Sci. (2013) 36:36–42.

[6] Wolf H. (2015). Therapeut behandelt Behinderte mit Riesenschnecken. [Accessed May 12, 2020].

[7] Richter C. (2016). Riesenschneck am Arm: Wie Tiere heilen können. Die Presse. [Accessed May 12, 2020].

[8] Thomas S. (2013). Medicinal use of terrestrial molluscs (slugs and snails) with particular reference to their role in the treatment of wounds and other skin lesions. World wide wounds. [Accessed April 8, 2016].

[9] Benthem Jutting WSSv. On the anatomy of Achatina fulica (Ferussac). Treubia. (1951) 21:111–3.

[10] Cooper JE Knowler C. Snails and snail farming: an introduction for the veterinary profession. Vet Rec. (1991) 129:541–9. PMID: 1801403

[11] Ghose KC. The alimentary system of Achatina fulica. Trans Am Microsc Soc. (1963) 82:149–67. doi: 10.2307/3223991

[12] Ghose KC. Reproductive system of the snail Achatina fulica. Proc Zool Soc London. (1963) 140:681–95. doi: 10.1111/j.1469-7998.1963.tb01993.x

[13] Goncalves TD. Anatomia macroscopica e microscopicado sisterna reprodutor de escargots das especies Achatina fulica e Achatina monochromatica. Sao Paulo: Universidade de Sao Paulo. Faculdade de Medicina Veterinaria e Zootecnia (2003).

[14] Teixeira DG Martinsi MF Guerra JL Blazquez FJH Sinhorini IL. Descrição histológica da via genital masculina e hermafrodita de escargots das espécies Achatina fulica e Achatina monochromatica. Braz J Vet Res Anim Sci. (2008) 45:413–20.

[15] Kumprataung W Kruatrachue M Suchart Upatham E Chitramvong Y Setarugsa P Chavadej J. Comparative studies on reproductive systems of Achatina fulica, Hemiplecta distincta and Cyclophorus aurantiacus. J Sci Soc Thail. (1989) 15:071–107. doi: 10.2306/scienceasia1513-1874.1989.15.071

[16] Smolowitz RB Lewbart GA. Gastropods In: Lewbart GA, editor. Invertebrate Medicine. Edn. Hoboken, NJ, USA: Wiley-Blackwell (2022). 151–75.

[17] Braun MH Chitty J. Clinical techniques of invertebrates. Vet Clin Exot Anim. (2006) 9:205–21. doi: 10.1016/j.cvex.2006.02.001 16759944

[18] Chitty J. Invertebrate medicine for the general practitioner. In Pract. (2015) 37:171–80. doi: 10.1136/inp.h422

[19] Pizzi R. 21: invertebrates In: Meredith A Johnson-Delaney C, editors. BSAVA manual of exotic pets. Edn. Gloucester, UK: BSAVA (2010).

[20] Frye FL. Pulmonates In: Frye FL, editor. Captive invertebrates. Malabar, Florida, USA: Krieger Publishing Company (1992). 75–8.

[21] Nollens HH Schofield JC Keogh JA Probert PK. Evaluation of radiography, ultrasonography and endoscopy for detection of shell lesions in live abalone, Haliotis iris (Mollusca: Gastropoda). Dis Aquat Org. (2002) 50:145–52. doi: 10.3354/dao050145, PMID: 12180705

[22] Walker A Ablett JD Sykes D Rücklin M Breure ASH. (2014). Using micro-CT for 3D visualisations of terrestrial snail soft tissue anatomy. Poster “Molluscan Forum.” 20 November London.

[23] Whisson CS Breure ASH. A new species of Bothriembryon (Mollusca, Gastropoda, Bothriembryontidae) from South-Eastern Western Australia. ZooKeys. (2016) 581:127–40. doi: 10.3897/zookeys.581.8044 PMC4857043 27199583

[24] Metscher BD. MicroCT for comparative morphology: simple staining methods allow high-contrast 3D imaging of diverse non-mineralized animal tissues. BMC Physiol. (2009) 9:11. doi: 10.1186/1472-6793-9-11, PMID: 19545439 PMC2717911

[25] Walczak M Binkowski M Sulikowska-Drozd A Wróbel Z. Maximum sphere method for shell patency measurements in viviparous land snails based on X-ray microcomputed tomography imaging. Comput Biol Med. (2015) 64:187–96. doi: 10.1016/j.compbiomed.2015.06.004, PMID: 26189157

[26] Penney BK Ehresmann KR Jordan KJ Rufo G. Micro-computed tomography of spicule networks in three genera of dorid sea-slugs (Gastropoda: Nudipleura: Doridina) shows patterns of phylogenetic significance. Acta Zool. (2020) 101:5–23. doi: 10.1111/azo.12266

[27] Chen C Copley JT Linse K Rogers AD Sigwart JD. The heart of a dragon: 3D anatomical reconstruction of the ‘scaly-foot gastropod’ (Mollusca: Gastropoda: Neomphalina) reveals its extraordinary circulatory system. Front Zool. (2015) 12:13. doi: 10.1186/s12983-015-0105-1, PMID: 26085836 PMC4470333

[28] Faulwetter S Vasileiadou A Kouratoras M Thanos D Arvanitidis C. Micro-computed tomography: introducing new dimensions to taxonomy. ZooKeys. (2013) 263:1–45. doi: 10.3897/zookeys.263.4261, PMID: 23653515 PMC3591762

[29] Postnov A De Clerck N Sasov A Van Dyck D. 3D in-vivo X-ray microtomography of living snails. J Microsc. (2002) 205:201–4. doi: 10.1046/j.0022-2720.2001.00986.x, PMID: 11879434

[30] Cooper JE. Anesthesia, analgesia, and euthanasia of invertebrates. ILAR J. (2011) 52:196–204. doi: 10.1093/ilar.52.2.196, PMID: 21709312

[31] Ziegler A Bock C Ketten DR Mair RW Mueller S Nagelmann N. Digital three-dimensional imaging techniques provide new analytical pathways for malacological research. Am Malacol Bull. (2018) 36:248–73. doi: 10.4003/006.036.0205

[32] Odaibo AO, (2019). Shell morphology, Radula and genital structures of new invasive Giant African land snail species, Achatina fulica Bowdich, 1822, Achatina albopicta E.A. smith (1878) and Achatina reticulata Pfeiffer 1845 (Gastropoda: Achatinidae) in Southwest Nigeria. bioRxiv.

[33] Zachariah TM. Chapter 3: invertebrates In: Mitchell MAT Th N, editors. Manual of exotic pets. St. Louis, Missouri: Saunders (2009)

[34] Morton JEY. Classification and structure of Mollusca In: Wilbur KMY, editor. Physiology of Mollusca, vol. I. New York and London: Academic Press (1964). 1–57.

[35] Davis R Chitty JR Saunders RA. (2001). Cardiovascular monitoring of an Achatina snail with a Doppler ultrasound probe. Proc Brit Vet Zool Soc, Autumn Meeting. 101.

[36] McConnachie S Alexander GJ. The effect of temperature on digestive and assimilation efficiency, gut passage time and appetite in an ambush foraging lizard, Cordylus melanotus melanotus. J Comp Physiol B. (2004) 174:99–105. doi: 10.1007/s00360-003-0393-1 14598178

[37] Bullough WS. Order Pulmonata - Genus Helix In: Bullough WS, editor. Practical invertebrate anatomy. London, UK: The MacMillan Press Ltd (1958). 363–9.

[38] Martin AW Stewart DM Harrison FM. Urine formaion in a pulmonate land snail, Achatina fulica. J Exp Biol. (1965) 42:99–123. doi: 10.1242/jeb.42.1.99 14294948

[39] Schmidt-Ukaj SG Gumpenberger M Mutschmann F Richter B. Case report: four cases of kidney disease in Giant African land snails (Lissachatina fulica). Front Vet Sci. (2023) 10:1152281. doi: 10.3389/fvets.2023.1152281 37255999 PMC10225638

[40] Guallart J Ferranti MP Bacigalupo L Chiantore M. In vivo magnetic resonance imaging to assess the sexual maturity of the endangered limpet Patella ferruginea. J Molluscan Stud. (2020) 86:422–6. doi: 10.1093/mollus/eyaa021

